# Two New Shrinking-Circle Methods for Source Localization Based on TDoA Measurements

**DOI:** 10.3390/s18041274

**Published:** 2018-04-20

**Authors:** Mingzhi Luo, Xiang Chen, Shuai Cao, Xu Zhang

**Affiliations:** Department of Electronic Science and Technology, University of Science and Technology of China (USTC), Hefei 230026, China; lmz2514@mail.ustc.edu.cn (M.L.); caoshuai@ustc.edu.cn (S.C.); xuzhang90@ustc.edu.cn (X.Z.)

**Keywords:** target localization, TDoA, shrinking circle method, dichotomy

## Abstract

Time difference of arrival (TDoA) measurement is a promising approach for target localization based on a set of nodes with known positions, with high accuracy and low complexity. Common localization algorithms include the maximum-likelihood, non-linear least-squares and weighted least-squares methods. These methods have shortcomings such as high computational complexity, requiring an initial guess position, or having difficulty in finding the optimal solution. From the point of view of geometrical analysis, this study proposes two new shrinking-circle methods (SC-1 and SC-2) to solve the TDoA-based localization problem in a two-dimensional (2-D) space. In both methods, an optimal radius is obtained by shrinking the radius with a dichotomy algorithm, and the position of the target is determined by the optimal radius. The difference of the two methods is that a distance parameter is defined in SC-1, while an error function is introduced in SC-2 to guide the localization procedure. Simulations and indoor-localization experiments based on acoustic transducers were conducted to compare the performance differences between the proposed methods, algorithms based on weighted least-squares as well as the conventional shrinking-circle method. The experimental results demonstrate that the proposed methods can realize high-precision target localization based on TDoA measurements using three nodes, and have the advantages of speed and high robustness.

## 1. Introduction

Target localization based on a set of nodes with known positions has received considerable interest in recent years due to its various applications in wireless communication, navigation, surveillance, and teleconferencing [1,2,3,4]. Typically, there are two working modes in real-world scenarios. In one mode, the target emits a signal while nodes receive the signal. In another mode, the nodes emit signals that are detected by the target. The measurements commonly used in target localization include the signal’s direction of arrival (DoA), received signal strength (RSS), time of arrival (ToA), time difference of arrival (TDoA) [5], and frequency difference of arrival (FDoA) [6,7,8].

The DoA technique estimates position by measuring the direction of the target relative to the fixed nodes. The RSS approach estimates the distance between the target and nodes by measuring the energy of the received signal. The ToA and TDoA methods estimate the distance using measurements of the travel times and the difference of travel times, respectively. TDoA is considered a promising approach due to its high accuracy and low complexity. TDoA positioning generally achieves higher localization accuracy than RSS and DoA [9,10]. Compared to the ToA method needing synchronization among the nodes and the target, only nodes need to be synchronized in a TDoA-based system. The FDoA technique measures the relative velocity between the target and the sensors, and might be a complementary method to TDoA. The joint usage of TDoA and FDoA can estimate the source position and velocity accurately when the source is moving [11], and has attracted a lot of research interest in the fields of surveillance [12], navigation [13], wireless communications [14] and sensor networks [15].

The problem of TDoA-based target localization is formulated as an optimization problem, which is not easy to resolve because the optimization objective is non-convex. Many methods have been proposed to solve the optimization problem. The maximum-likelihood (ML) method has been considered as an optimal and robust method for parameter estimation [16]. ML searches for the optimal solution in a global area; however, it involves high computational complexity, and obtaining the optimal solution is not guaranteed [17]. The non-linear least-squares (NLS) approach based on Taylor-series expansion has also been applied to parameter estimation [18]. The drawback of the NLS method is that an initial guess position, which may influence performance, is needed. The closed-form weighted least-squares (WLS) algorithm has been verified to be effective in target localization by introducing an additional variable to rearrange the non-linear equations into linear equations [19]. To improve further the localization performance of the WLS method, two-step weighted least-squares (2WLS) [20] and constrained weighted least-squares (CWLS) [21] have been put forward successively. The performance of 2WLS and CWLS decreases when the target is approaching the center because the system matrix related to the linear equations is ill-conditioned. A new CWLS estimator called separated CWLS (SCWLS) was proposed and proved to be effective in solving this problem [22]. However, due to the non-convex nature of CWLS, it is hard to obtain a global optimal solution, and the problem was reformulated as a convex optimization problem by exploiting the hidden convexity [23].

In summary, localization algorithms based on WLS are founded upon optimal parameter estimation. By introducing an additional variable or exploiting the hidden relationships among the variables, different WLS-based methods achieve the Cramér–Rao lower bound under some mild approximations. The closed-form localization algorithms require at least four nodes if they do not lie on a straight line for 2-D localization [24]. Nevertheless, there are some problems that need to be considered in a real-world positioning system. Taking an indoor localization system based on audible sound as an example, the quantity of nodes is limited by the refresh rate and budget. Therefore, how to achieve high-precision positioning using fewer nodes is a problem that needs to be considered. In addition, accurate localization under the phenomenon of non-line-of-sight (NLOS) is also a great challenge.

Some efforts have also been made to solve the source localization problem from the point of view of geometrical analysis. Generally, localization algorithms based on geometric methods need fewer nodes to locate the target position. Because each pair of nodes and their time difference determines a hyperbola, conventional localization methods based on geometrical analysis are usually based on the idea of finding the point at which these hyperbolic lines intersect [25]. For instance, one method proposed in [26] is based on the idea that three nodes and their set of time differences determine the major axis of an ellipse. When there are more than three nodes, there will be several major axes. An estimation of the target is the point at which these major axes intersect. The procedure of computing the intersections is thought to be quite time-consuming. A representative source localization method based on geometric analysis is the shrinking-circle method. The shrinking-circle method was first proposed in [27] and the idea is to shrink the radius of all circles at a constant step length until the intersection area reaches a small threshold. This method was also mentioned in the work of [28], which points out that performing “circle shrinking” can be computationally demanding. In [29], an improved version, which searches for the optimal radius with a large step length at first and then reduces the step length to obtain a more accurate solution, was explored. Apart from the related works, two new shrinking-circle schemes that employ a dichotomy strategy are proposed in this study. Taking the target localization with a network of static nodes employing TDoA measurement as the application object, the performance of the two proposed methods are verified by simulation and indoor-positioning experiments.

The rest of the paper is organized as follows. In Section 2, the idea of the shrinking-circle method is reviewed, and then the two new shrinking-circle schemes (SC-1 and SC-2) are introduced. Subsequently, simulation and indoor-localization experiment results of the proposed methods, algorithms based on weighted least-squares as well as the conventional shrinking-circle method, are presented and compared in Section 3 to demonstrate the performance of the proposed methods. Finally, the conclusion and discussion are given in Section 4.

## 2. Shrinking-Circle Method Based on Time Difference of Arrival (TDoA)

In this section, the idea of shrinking-circle method based on TDoA is described. And then, two shrinking-circle schemes employing a dichotomy strategy are proposed.

### 2.1. The Idea of the Shrinking-Circle Method

**Figure 1:**
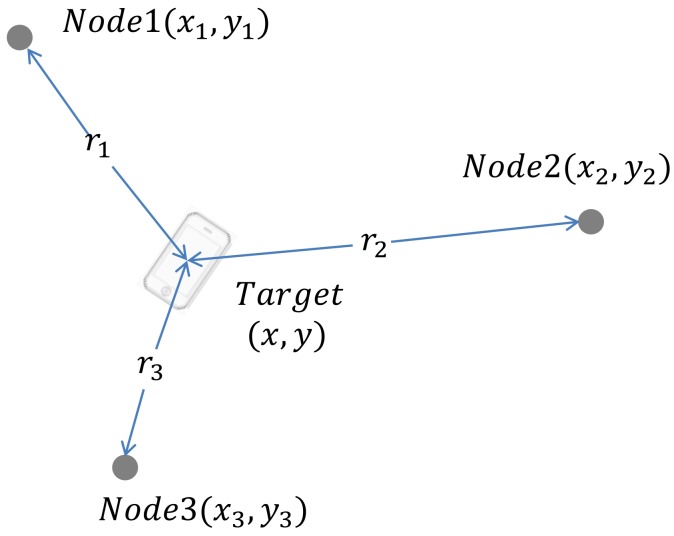
Schematic diagram of target localization.

Considering the positioning system shown in Figure 1, the target localization based on TDoA measurement can be defined as below. Let $N_{i}\left(x_{i},y_{i}\right)$ denote the coordinate of the ith node, T(x,y) denote the target’s coordinate, and $r_{i}$ represent the distance between the target and the ith node. The TDoA between nodes i and j can be computed according to Equations (1)–(3):

(1) $${\left(x-x_{i}\right)}^{2}+{\left(y-y_{i}\right)}^{2}=r_{i}^{2}$$

(2) $$d_{ij}=r_{j}-r_{i}\;\left(i,j=1,\;\cdots ,n\right)$$

(3) $$TDoA_{ij}=d_{ij}/c$$

where n is the quantity of nodes, and c represents the speed of propagation. Taking the 1st node as a reference, $TDoA_{1i}$ can be measured by the system and be used to compute $d_{1i}$ according to Equation (3). The goal of target localization based on TDoA measurement is to find the optimal (x,y) that minimizes the error function f(x,y) (Equation (4)). The problem of TDoA-based localization is then formulated as an optimization problem.


(4) $$f\left(x,y\right)=\overset{n}{\underset{i=1}{\sum }}{\left(\sqrt{{\left(x-x_{i}\right)}^{2}+{\left(y-y_{i}\right)}^{2}}-\sqrt{{\left(x-x_{1}\right)}^{2}+{\left(y-y_{1}\right)}^{2}}-d_{1i}\right)}^{2}$$

Traditionally, a two-dimensional search algorithm is applied to find the optimal solution.

Because there are two variables in the equation (x and y), two or more equations need to be found. Consequently, at least three nodes are needed to generate enough range difference $d_{ij}$ to establish the equations.

**Figure 2:**
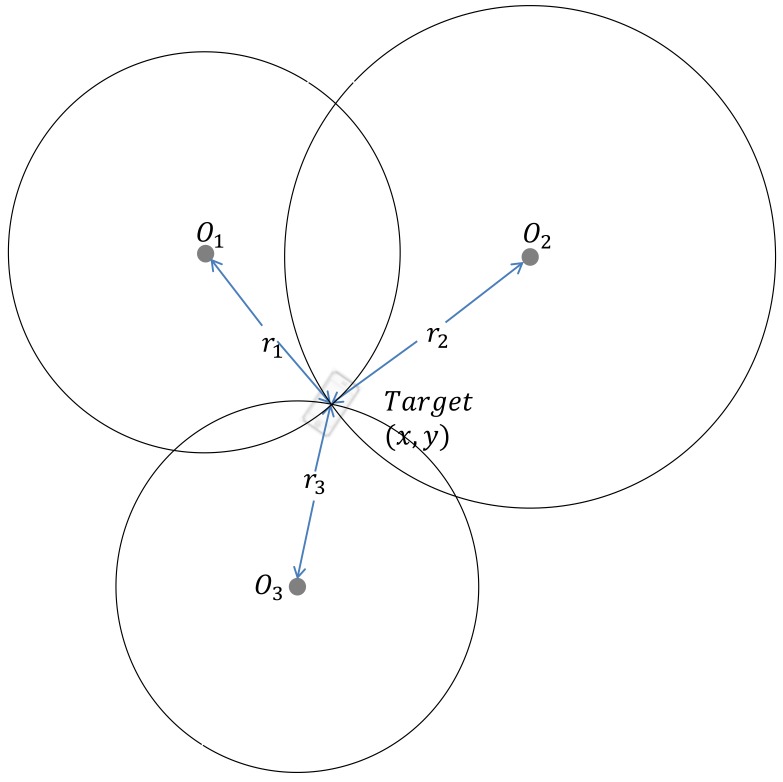
The basic idea of the shrinking-circle method.

From Equation (1), it is easy to find that the target T is on the circumference of a circle with $N_{i}$ as the center with radius $r_{i}$. The basic idea of the shrinking-circle method is to find the perfect radius $r_{i}$ with which all the circles intersect at the same point, as shown in Figure 2. The solution of the equation is then the coordinates of the intersection point.

Taking the 1st node as a reference, the radius of circle $O_{i}$ could be described as Equation (5), and Equation (1) can be converted to Equation (6):

(5) $$r_{i}=r_{1}+d_{1i}$$

(6) $${\left(x-x_{i}\right)}^{2}+{\left(y-y_{i}\right)}^{2}={\left(r_{1}+d_{1i}\right)}^{2}$$

Because $d_{1i}$ can be computed from TDoA values, the radius $r_{1}$ is the only variable to be considered in Equation (6). The traditional two-dimensional search algorithm is changed to a one-dimensional search algorithm by this method. One strategy is to shrink the radius of all circles at a constant step length until the intersection area reaches a small threshold [27]. In other work [29], the conventional shrinking-circle method (CSC) searches for the optimal radius with a large step length at first and then reduces the step length to obtain a more accurate solution. In this paper, a dichotomy strategy is applied to reduce the computational complexity, and a localization system with only three nodes was used to show how the strategy works.

The distance between node $N_{i}$ and $N_{j}$ is defined as Equation (7). When the circle $O_{i}$ intersects with circle $O_{j}$, the radii of the two circles should fit Equations (8) and (9):

(7) $$L_{ij}=L_{ji}=\sqrt{{\left(x_{i}-x_{j}\right)}^{2}+{\left(y_{i}-y_{j}\right)}^{2}}$$

(8) $$r_{i}+r_{i}+d_{ij}\geq L_{ij}$$

(9) $$r_{i}\geq \left(L_{ij}-d_{ij}\right)/2$$

The target should be located near the triangular area formed by three nodes. It is assumed that the target is in the area surrounded by the nodes, so the maximum radius of circle $N_{i}$ should satisfy Equation (10):

(10) $$r_{i}\leq max\{L_{ij}\}$$

Therefore, the basic radius $r_{1}$ should fulfill Equation (11):

(11) $$\begin{array}{c} R_{min}\leq {\;r}_{1}\;\leq R_{max}\\ R_{min}=min\left\{\left(L_{1j}-d_{1j}\right)/2\right\}\\ R_{max}=max\left\{L_{1j}\right\} \end{array}$$

### 2.2. The First Shrinking-Circle Method Employing the Dichotomy

**Figure 3:**
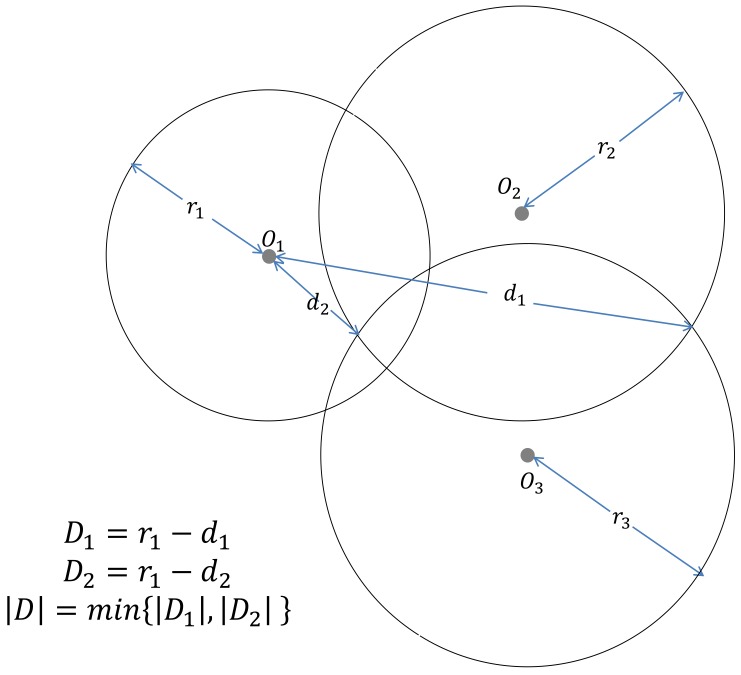
Definition of the value of D using circle $O_{1}$ as a reference.

**Figure 4:**
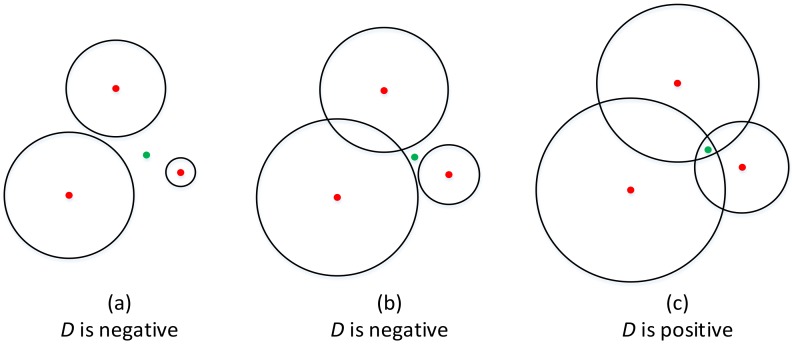
The sign of D depends on different conditions.

In the first method (SC-1), a distance parameter D is defined to guide the localization procedure. In the localization system with three nodes, D is defined as the minimum distance of the intersections of two circles to the circumference of the third circle (Figure 3). The sign of D depends on the following three conditions (Figure 4):

(a) When there are no intersections, D is a constant negative number.

(b) When both of the intersections are in or out of the third circle, D is negative.

(c) When one of the intersections is in the third circle while another is out of the circle, D is positive.

Condition (c) can be fulfilled when the radii are big enough, and (a) is fulfilled when the radii are small enough.

Based on distance D and Equation (11), the dichotomy algorithm can be applied in this situation to search for the perfect radius $r_{1}$ using the following procedure:

Step 1. Compute the maximum radius $R_{max}$ and minimum radius $R_{min}$ of $r_{1}$.

Step 2. Compute $R_{mid}$, where $R_{mid}=\left(R_{max}+R_{min}\right)/2$.

Step 3. Calculate the distance D when $r_{1}$ equals $R_{mid}$.

Step 4. Update the value of $R_{max}$ and $R_{min}$. If D is positive, $R_{max}=R_{mid}$; otherwise, $R_{min}=R_{mid}$.

Step 5. Compare the value of $\left|R_{max}-R_{min}\right|$ with a threshold TH. If it is larger than TH, return to Step 2; otherwise, terminate the procedure, and the value of $R_{mid}$ is considered as the optimum radius.

When the optimum radius of $r_{1}$ is known, the coordinate of the target can be obtained by the following steps:

Step 1. Calculate the coordinate of all the intersections of three circles.

Step 2. Obtain the absolute value of D corresponding to each intersection and remove the intersections with an absolute value of D that is larger than the average of D to simplify the following step.

Step 3. Calculate the distance between each pair of the remaining intersections, and the position of the target is the midpoint of the closest pair of intersections.

### 2.3. The Second Shrinking-Circle Method Employing the Dichotomy

In the second method (SC-2), an error function $f\left(r_{1}\right)$ is introduced to guide the positioning based on the following reasoning process.

Firstly, set i=1 and 2 in Equation (6) to obtain Equations (12) and (13).


(12) $${\left(x-x_{1}\right)}^{2}+{\left(y-y_{1}\right)}^{2}=r_{1}^{2}$$

(13) $${\left(x-x_{2}\right)}^{2}+{\left(y-y_{2}\right)}^{2}={\left(r_{1}+d_{12}\right)}^{2}$$

Then, obtain the Equation (14) by subtracting (12) from (13). Equation (14) is a linear equation that represents the straight line that goes through the intersections of circle $O_{1}$ and circle $O_{2}$.


(14) $$\left(x_{1}-x_{2}\right)x+\left(y_{1}-y_{2}\right)y=\left(d_{12}^{2}+2r_{1}d_{12}+x_{1}^{2}-x_{2}^{2}+y_{1}^{2}-y_{2}^{2}\right)/2$$

Similarly, set i=1 and 3 in Equation (6) and obtain Equation (15).


(15) $$\left(x_{1}-x_{3}\right)x+\left(y_{1}-y_{3}\right)y=\left(d_{13}^{2}+2r_{1}d_{13}+x_{1}^{2}-x_{3}^{2}+y_{1}^{2}-y_{3}^{2}\right)/2$$

By combining (14) and (15), a solution can be obtained as shown in Equation (16).

(16) $$X=A^{-1}H$$

where X=[x,y]’, $A=\left(\begin{array}{cc} x_{1}-x_{2} & y_{1}-y_{2}\\ x_{1}-x_{3} & y_{1}-y_{3} \end{array}\right)$, $H=\frac{1}{2}\left(\begin{array}{c} d_{12}^{2}+2r_{1}d_{12}+x_{1}^{2}-x_{2}^{2}+y_{1}^{2}-y_{2}^{2}\\ d_{13}^{2}+2r_{1}d_{13}+x_{1}^{2}-x_{3}^{2}+y_{1}^{2}-y_{3}^{2} \end{array}\right)$.

Thus, the solution X is determined by the only variable $r_{1}$, and the only task is to find the optimal value of $r_{1}$.

The error function $f\left(r_{1}\right)$ is defined as Equation (17). According to the definition, it is obvious that the optimal value of $r_{1}$ satisfies Equation (18).


(17) $$f\left(r_{1}\right)=\overset{n}{\underset{i=1}{\sum }}\left({\left(x-x_{i}\right)}^{2}+{\left(y-y_{i}\right)}^{2}-{\left(r_{1}+d_{1i}\right)}^{2}\right)$$

(18) $$f\left(r_{1}\right)=0$$

Although it is not easy to solve Equation (18) directly, the solution of (18) within the range defined by (11) is unique in most of the localization area. Thus, the dichotomy algorithm can be applied to obtain the unique solution, and the procedure involves the following steps:

Step 1. Compute the maximum radius $R_{max}$ and minimum radius $R_{min}$ of $r_{1}$.

Step 2. Compute $R_{mid}$, where $R_{mid}=\left(R_{max}+R_{min}\right)/2$.

Step 3. Calculate the solution X when $r_{1}$ equals $R_{mid}$, and then calculate $f\left(r_{1}\right)$.

Step 4. Update the value of $R_{max}$ and $R_{min}$. If $f\left(r_{1}\right)>0$, $R_{min}=R_{mid}$; otherwise, $R_{max}=R_{mid}$.

Step 5. Compare the value of $\left|f\left(r_{1}\right)\right|$ with a threshold $TH_{1}$ and compare the value of $\left|R_{max}-R_{min}\right|$ with a threshold $TH_{2}$. If $\left|f\left(r_{1}\right)\right|<TH_{1}$ or $\left|R_{max}-R_{min}\right|<TH_{2}$, terminate the procedure; otherwise, return to Step 2. When the procedure is finished, X is the coordinate of the target.

### 2.4. Localization Error Parameters

The performance of the two proposed SC methods was compared to SCWLS [22], CWLS [21], 2WLS [20], and the CSC [29] methods. Simulations and indoor localization experiments based on acoustic transducers were conducted. The performance of the localization methods was evaluated by localization error Er and the mean localization error (MLE). The localization error is defined as Equation (19):

(19) $$Er=\|\;u-\hat{u}\|$$

where u denotes the true position, and $\hat{u}$ is the estimate of the true position. The MLE is computed using N independent estimations as Equation (20).

(20) $$MLE=\frac{1}{N}\sum^{\text{​}}\|u\left(i\right)-\hat{u}\left(i\right)\|$$

where $\hat{u}\left(i\right)$ is the estimate of u(i) at the ith estimation.

## 3. Results

Simulations and indoor-localization experiments based on acoustic transducers were conducted in this section, and the results of the proposed methods, algorithms based on weighted least-squares as well as the conventional shrinking-circle method are presented and compared.

### 3.1. Simulation Experiment

**Figure 5:**
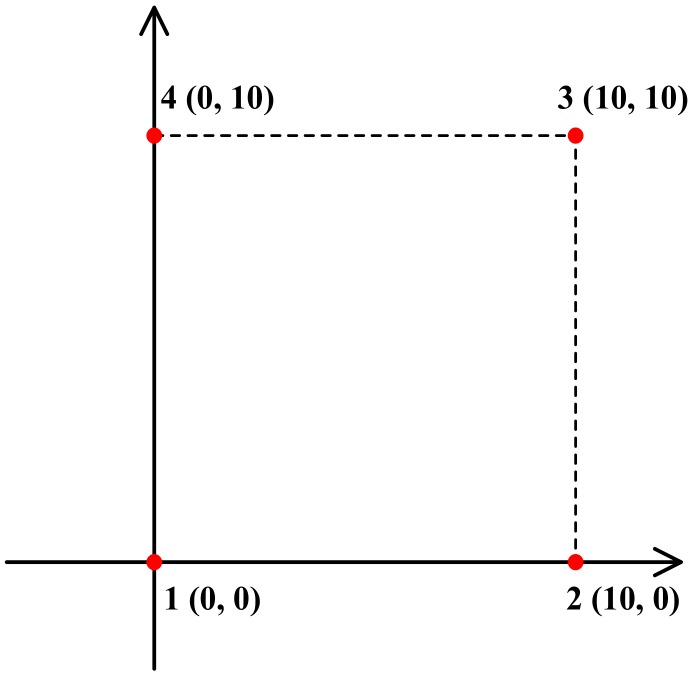
The layout of four nodes in the simulation experiment.

The localization simulation experiment was done using Matlab R2015a. All results were obtained using the same computer with a 3.60 GHz CPU and 8 GB of RAM. The range difference was used instead of TDoA, which can be obtained by dividing the range difference by the speed of propagation. Four nodes were applied in the simulation, and the layout of the nodes is shown in Figure 5. The coordinates of the four nodes are 1 (0, 0), 2 (10, 0), 3 (10, 10), and 4 (0, 10). 251 × 251 points were selected evenly throughout the whole experiment area.

#### 3.1.1. Properties of Method SC-1

**Figure 6:**
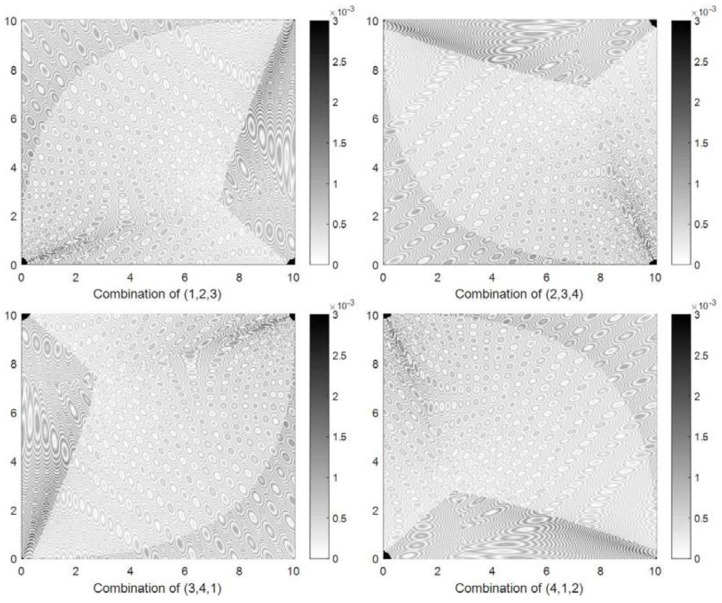
Four localization error distributions obtained by method SC-1 in the simulation experiment.

Shrinking circle methods can locate a target using three nodes, so four localization error distributions were obtained using four three-node combinations (1, 2, 3), (2, 3, 4), (3, 4, 1), and (4, 1, 2). Figure 6 shows the localization error distribution obtained by method SC-1. A localization error that is larger than $3\times 10^{-3}$ is indicated by the darkest color. It can be observed that different three-node combinations obtain different localization error distributions. The localization errors are lower than $1.5\times 10^{-3}$ in most of the experimental area but are much larger than $3\times 10^{-3}$ in the areas close to the nodes.

**Figure 7:**
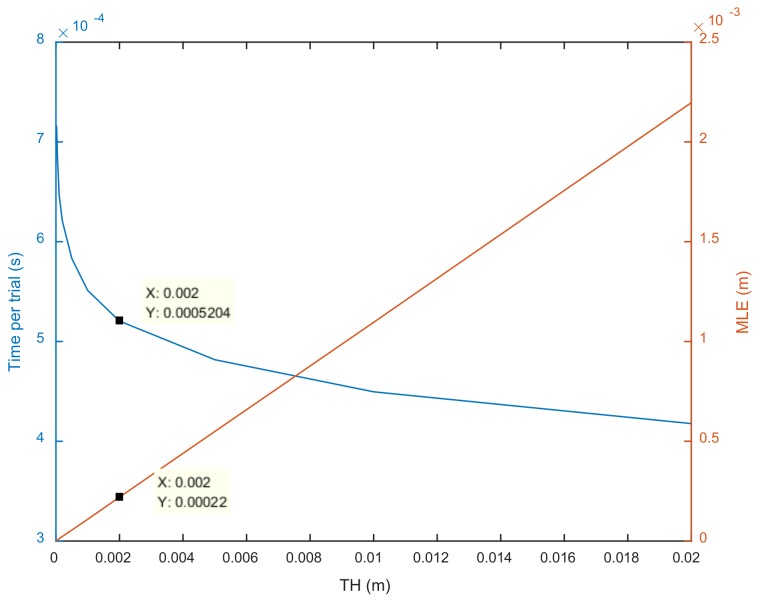
Influence of threshold TH on the required time and mean localization error MLE.

Figure 7 shows the influence of threshold TH on MLE and the time needed for each localization trial. The time needed for each trial has an exponential relation with TH, while the relationship between MLE and TH is linear. To obtain the best accuracy without requiring too much time, the value of TH was finally set to 0.002 after considering the accuracy, efficiency, and practical localization requirements. Under this condition, the MLE of four combinations is $2.2\times 10^{-4}$, and the computation time per trial is $5.20\times 10^{-4}$s for method SC-1. The computational complexity of the SC-1 is roughly $O\left(14\left(Nn+Nn^{2}+nN^{2}\right)\right)$ (where N is the dimension of the source location and *n* is the number of nodes).

#### 3.1.2. Properties of Method SC-2

**Figure 8:**
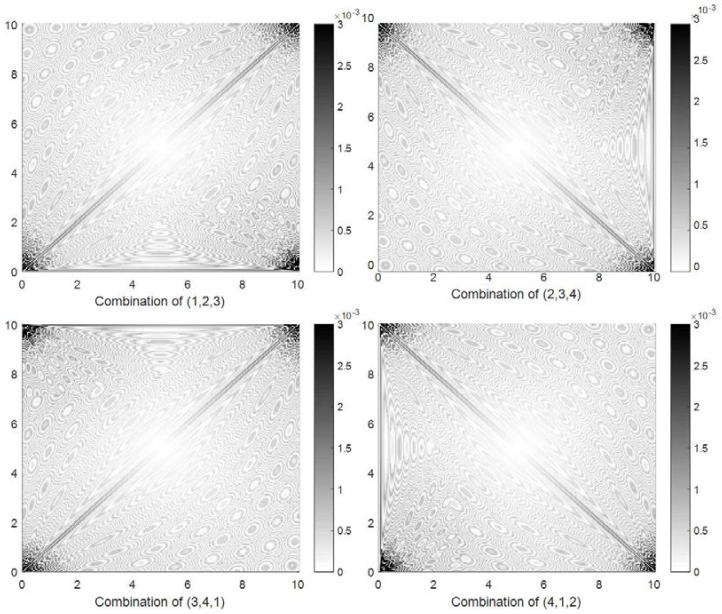
Four localization error distributions obtained by method SC-2 in the simulation experiment.

Figure 8 shows the localization error distribution obtained by method SC-2. Similar to method SC-1, different three-node combinations obtain different localization error distributions. However, this method is superior to method SC-1: the localization errors are less than $1\times 10^{-3}$ in most of the area and around $3\times 10^{-3}$ near the nodes. The minimum positioning error was obtained in the center, but the maximum error appeared in the area near the nodes. Consider the combination of (1, 2, 3) as an example. The localization errors are larger in the areas near node 1, node 2, and node 3 than in other areas.

**Figure 9:**
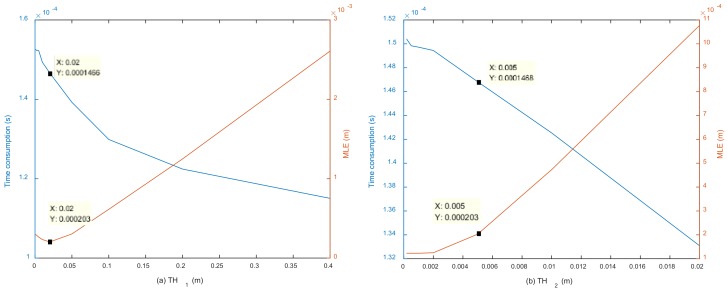
(**a**) Influence of $TH_{1}$ on the required time and MLE with $TH_{2}=0.005$; (**b**) Influence of $TH_{2}$ on the required time and MLE with $TH_{1}=0.02$.

Figure 9 shows the influence of thresholds $TH_{1}$ and $TH_{2}$ on MLE and the time required in each trial. Both $TH_{1}$ and $TH_{2}$ have linear relations with the required time and have a relatively small impact on it. $TH_{1}$ was set to 0.02, and $TH_{2}$ was set to 0.005 after considering both accuracy and efficiency. Under this condition, the MLEs of the four combinations are $2.03\times 10^{-4}$, and the computation time per trial is $1.47\times 10^{-4}$s for method SC-2. Under this condition, the computational complexity of SC-2 is around $O\left(10\left(Nn^{2}+nN^{2}\right)\right)$.

#### 3.1.3. Localization Error Distributions of Other Methods

**Figure 10:**
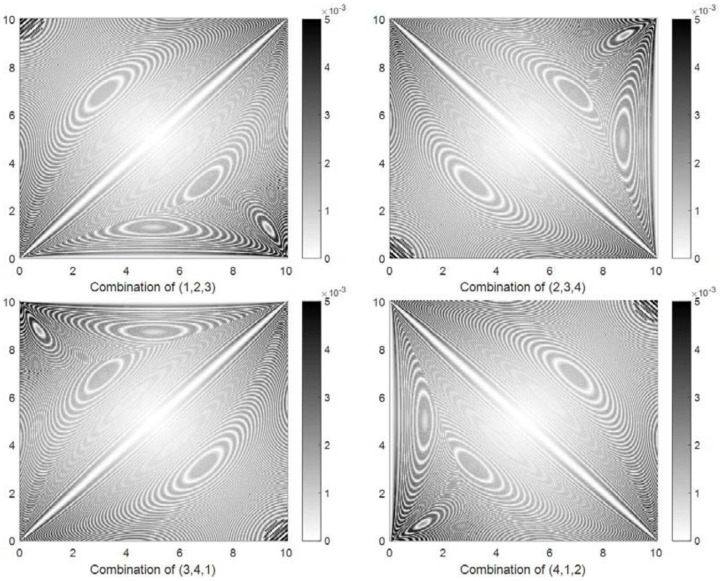
Four localization error distributions obtained by the conventional shrinking-circle (CSC) method.

Figure 10 shows the localization error distributions obtained by the CSC method. Similar to the proposed SC methods, different three-node combinations obtained different localization error distributions. In most of the area, the localization errors are lower than $3\times 10^{-3}$. In contrast to the proposed methods, the minimum positioning error was obtained on the diagonal of the two nodes, but the maximum error appeared in the areas near the other two nodes. Consider the combination of (1, 2, 3) as an example. The localization errors are larger in the areas near node 2 and node 4 than in other areas. The required time per localization trial is $2.11\times 10^{-4}$s for the CSC method.

**Figure 11:**
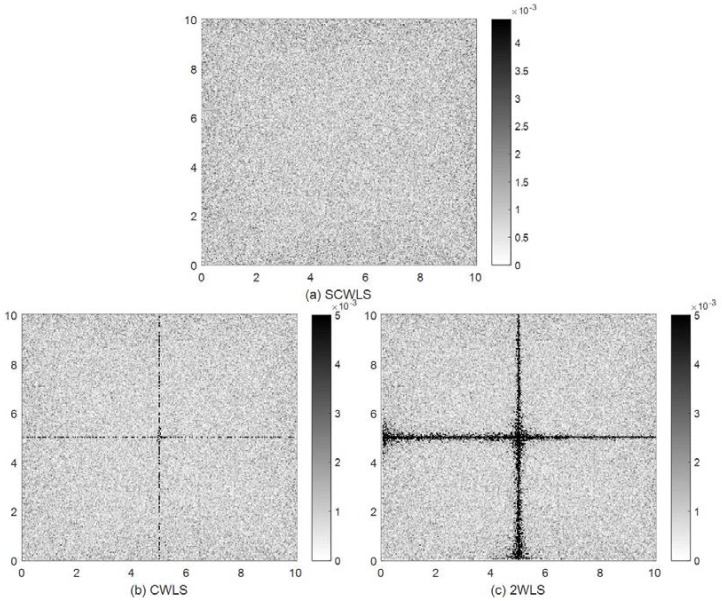
Localization error distributions obtained by (**a**) separated constrained weighted least-squares (SCWLS) method; (**b**) CWLS method; and (**c**) two-step weighted least-squares (2WLS) method.

Figure 11 shows the localization error distributions obtained by the SCWLS, CWLS and 2WLS methods. The localization errors are less than $2.5\times 10^{-3}$ in most of the experiment area for these three methods. Unlike the shrinking circle methods, the localization errors are evenly distributed in most of the area for the SCWLS, CWLS, and 2WLS methods. SCWLS obtained the most uniform error distribution. Both CWLS and 2WLS obtained large localization errors when the target was on or near the axis of symmetry of the experimental area. The required times per localization trial were $1.23\times 10^{-3}$s, $1.15\times 10^{-3}$s, and $2.61\times 10^{-4}$s for SCWLS, CWLS, and 2WLS, respectively.

#### 3.1.4. Robustness

A robustness experiment was carried out to assess the performance of the SC-1, SC-2, SCWLS, CWLS, 2WLS and CSC methods against different background noise. The noise factor β is defined by Equation (21), where $\sigma^{2}$ is the variance of zero mean Gaussian noise, which was directly added to the range difference $d_{ij}$:

(21) $$\beta =10{log}_{10}\left(\sigma^{2}\right)$$

**Figure 12:**
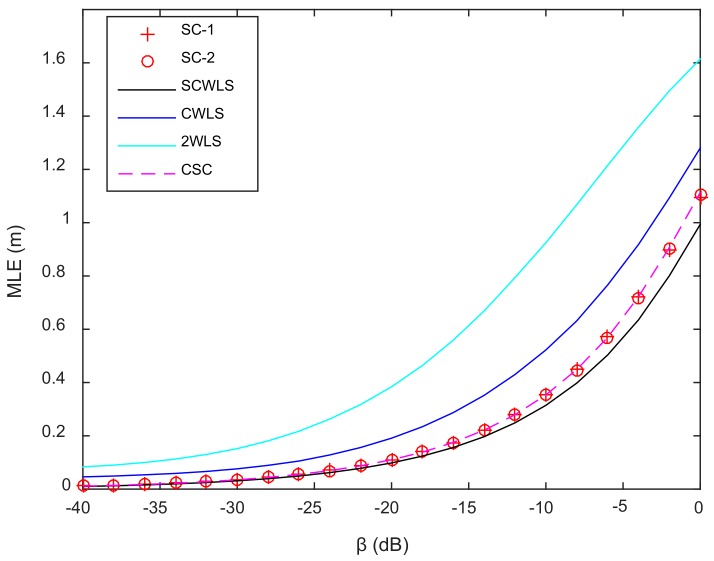
Relationship between β and MLE.

In the experimental area, 51 × 51 test points were evenly selected. On each test point, 100 independent localization trials were done to assess the robustness of the localization algorithms. Figure 12 shows the relationship between the noise factor β and MLE of different localization methods. CWLS and 2WLS performed poorly compared to the four other methods, while SCWLS had the best performance. For β<−25 dB, the MLEs of SC-1, SC-2, SCWLS and CSC were nearly the same. For β>−25 dB, SC-1, SC-2, and CSC had the same MLE, while SCWLS had a smaller MLE.

### 3.2. Indoor Localization Experiments Based on Acoustic Transducers

**Figure 13:**
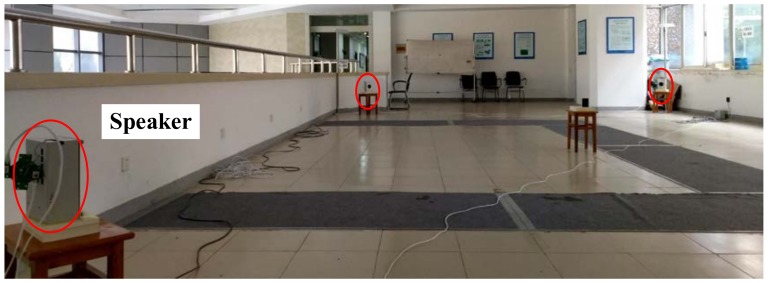
Experimental setup in an indoor environment.

An indoor localization system based on acoustic transducers was built to compare the localization performance of the different methods. Acoustic signals were employed to locate a cellphone when its user stands still at different points. Four speakers were deployed as nodes in a typical indoor environment, as shown in Figure 13. The speakers were organized in a rectangular shape (9.74 m by 6.09 m). The coordinates of the four speakers were 1 (0, 0), 2 (6.09, 0), 3 (6.09, 9.74), and 4 (0, 9.74). The cellphone was placed at the same height as the speakers. In this system, a signal-emitting scheme combining time-division multiplexing and frequency-division multiplexing was adopted. At the begin of each emitting cycle (1 s), two diagonal speakers emitted 50 ms-long different chirp signals at the same time, and the other two speakers emitted synchronously 50 ms-long different chirp signals at the 200th milliseconds. The processing of the acoustic signal and calculation of the TDoA information were undertaken on the phone. The TDoAs were saved to a text file and then input into the localization algorithms.

#### 3.2.1. Accuracy and Time Consumption

**Figure 14:**
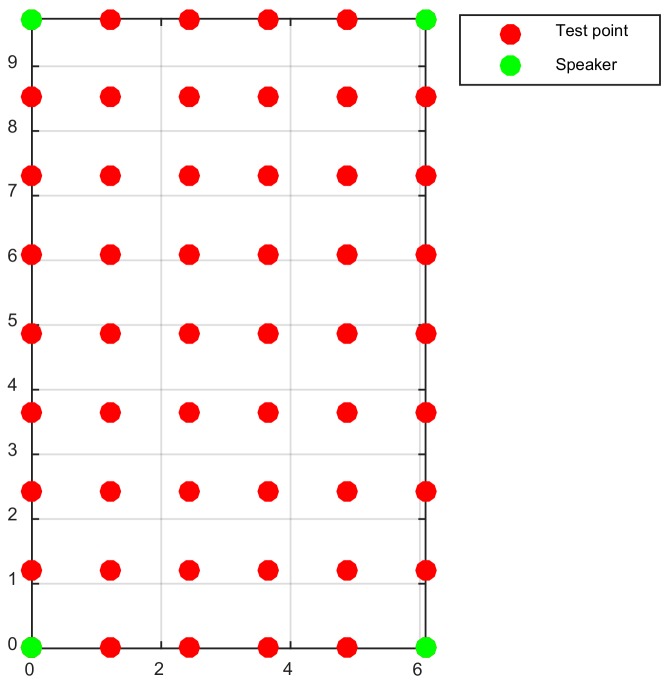
Sketch map of speakers and test points in the indoor localization system.

In this experiment, the 50 test points shown in Figure 14 were selected and tested. For each method, at least 65 localization trials were done at each point. The localization results of the shrinking-circle methods (SC-1, SC-2 and CSC) were the average results of four three-node combinations. Among the 3250 localization results, the trials with localization errors greater than 2 m were considered as bad results and removed from the result set.

**Figure 15:**
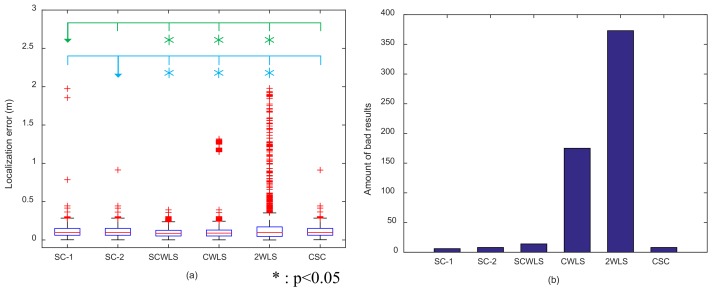
(**a**) Box plot of localization error of the six localization methods; (**b**) amount of bad results.

Figure 15 shows a box plot of the localization error and the amount of bad results of the six localization methods. CWLS and 2WLS performed poorly and had much larger outliers in terms of localization error and bad results than the other methods. The medians of localization error for the three shrinking-circle methods were around 0.097 m, and the amount of bad results was less than 15. SCWLS showed the best performance, and the median of localization error was 0.087 m.

A significance test was conducted between the proposed methods and the other four methods. In Figure 15a, the green line above the box plot represents the results compared with SC-1, and the blue line represents SC-2. The asterisk means there is a significant difference compared with SC-1 or SC-2. A *t*-test showed that both of the proposed methods showed significant differences with SCWLS, CWLS and 2WLS. However, there was no significant difference between the SC-1, SC-2, and CSC methods. The computation times per trial were $4.82\times 10^{-4}$s, $1.47\times 10^{-4}$s, $1.23\times 10^{-3}$s, $1.15\times 10^{-3}$s, $2.61\times 10^{-4}$s, and $2.11\times 10^{-4}$s for SC-1, SC-2, SCWLS, CWLS, 2WLS and CSC, respectively.

#### 3.2.2. Localization in Non-Line-of-Sight (NLOS) Environment

**Figure 16:**
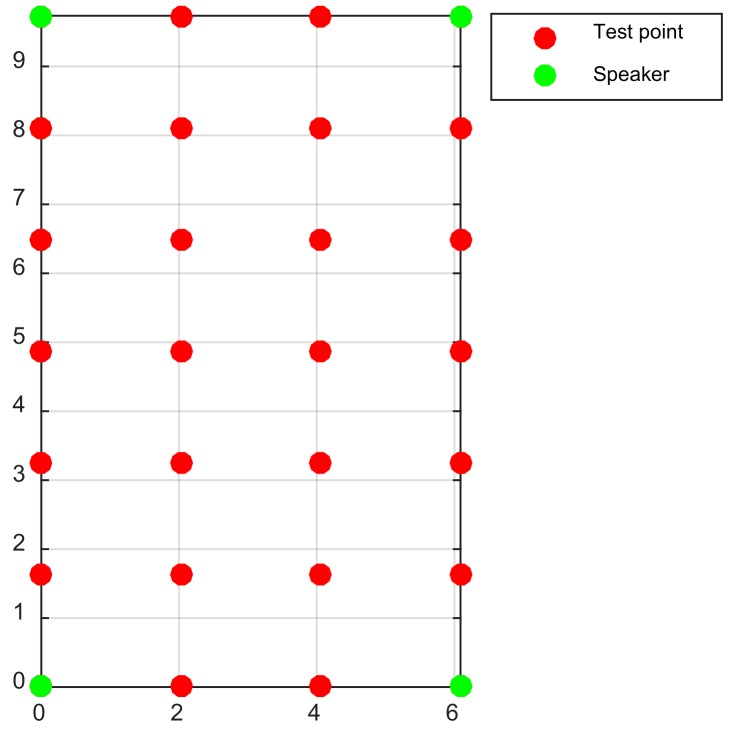
Sketch map of speakers and test points.

An experiment was conducted to verify the performance of the localization algorithms under the condition of NLOS. The experimental setup was the same as in Section 3.2.1, but the line of sight (LOS) between the cellphone and one of the speakers was blocked by the user’s body. There were 24 test points (as shown in Figure 16), and at each point each of four speakers was sheltered, respectively. At least 65 localization trials were conducted in each condition. Also, the trials with localization errors greater than 2 m were considered as bad results and removed from the result set.

**Figure 17:**
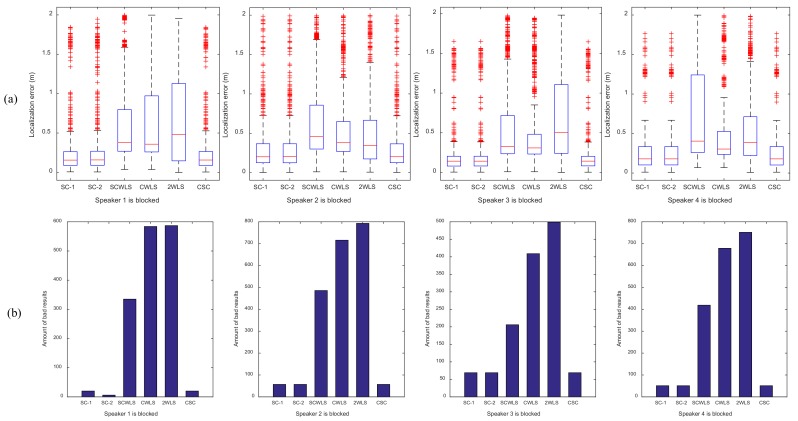
Experiment under the condition of one speaker being blocked. (**a**) Box plot of localization error; (**b**) amount of bad results.

The localization results of the shrinking circle methods (SC-1, SC-2, and CSC) are the results of the combinations of three speakers that were not blocked by the body. The other methods (SCWLS, CWLS and 2WLS) used all four speakers to locate the target. Figure 17 shows the box plot of the localization error and the amount of bad results of the six localization methods with one speaker being blocked. NLOS generated large localization errors, as expected. The median of localization error was around 0.15 m for the shrinking-circle methods (SC-1, SC-2 and CSC), but the error was greater than 0.30 m for the three other methods (SCWLS, CWLS and 2WLS). The amount of bad results is around 60 for the shrinking-circle methods, but it is much larger for the other methods. The SC-1, SC-2 and CSC methods had a similar performance to each other in the NLOS environment.

## 4. Conclusions and Discussion

This paper proposed two shrinking-circle methods that employ a dichotomy (SC-1 and SC-2) to solve the problem of target localization based on TDoA in a 2-dimensional space. The methods were compared to previous methods using simulations and indoor localization experiments, and the results showed the validity and limitations of the proposed methods.

As expected, the shrinking-circle methods needed fewer nodes to locate the target position compared with the SCWLS, CWLS and 2WLS algorithms. Based on knowing which node was blocked, the shrinking-circle methods showed an advantage during the experiment under the condition of NLOS. Additionally, compared with the SCWLS and CWLS methods, the three shrinking-circle methods took less time to locate the position of the target and showed better robustness than both the CWLS and 2WLS methods. However, SCWLS was a little more robust than all the shrinking-circle methods (SC-1, SC-2 and CSC).

SC-1 and SC-2 obtained a lower localization error than the CSC method when the target was not in an area near the nodes. However, for both the SC-1 and SC-2 methods, the localization accuracy declined when the target was near the nodes. The dichotomy strategy was employed to try to reduce the time required by the conventional shrinking circle (CSC) method [29]. However, the experimental results do not indicate the superiority of the proposed methods in this respect. Compared with the CSC method, SC-1 required more time, while SC-2 required less time in each localization trial. The process of computing the intersections in the SC-1 method did not employ matrix operations, while the SC-2 and CSC methods did. Because the matrix operations in Matlab were specially optimized to run faster, the SC-2 and CSC methods needed less time to locate the target than method SC-1. In fact, as shown in Section 3.1.1 and Section 3.1.2, the computational complexity of the SC-1 is roughly $O\left(14\left(Nn+Nn^{2}+nN^{2}\right)\right)$ (where N is the dimension of the source location and n is the number of nodes), and the computational complexity of SC-2 is about $O\left(10\left(Nn^{2}+nN^{2}\right)\right)$. Obviously, SC-2 has lower computational complexity than SC-1 method. In summary, the two proposed shrinking-circle methods could realize high-precision target localization based on TDoA measurement using three nodes. They also had the advantages of speed and high robustness. In future work, efforts should be made to solve the problem of localization near nodes and to achieve higher accuracy in locating a moving target.
